# Development of the Saudi Arabian trauma system

**DOI:** 10.25122/jml-2021-0066

**Published:** 2022-01

**Authors:** Sharfuddin Chowdhury, Dennis Mok, Luke Leenen

**Affiliations:** 1. Trauma Center, King Saud Medical City, Riyadh, Saudi Arabia; 2. Medical Management Consulting, Birkdale, Queensland, Australia; 3. Department of Trauma, University Medical Center Utrecht, Utrecht, Netherlands

**Keywords:** Saudi Arabia, growth and development, wounds and injuries, trauma system, emergency medical services, MVC – Motor vehicle crash, FOCUS-PDCA – Find, organize, clarify, understand, select-plan, do, check, and act, CAG – Clinical advisory group, VRO – Vision Realization Office, EMS – Emergency medical services, PHAP – Program of health assurance and purchase of health services, NTP – National Transformation Program, CBAHI – Central Board for Accreditation of Healthcare Institutions, SHC – Saudi Health Council, RTA – Road traffic accident, ACS-COT – American College of Surgeons Committee on Trauma

## Abstract

A dedicated network-based trauma system ensures optimal care to injured patients. Considering the significant burden of trauma, the Kingdom of Saudi Arabia is striving to develop a nationwide trauma system. This article describes the recent design, development, and implementation of the Saudi Arabian trauma system in line with Vision 2030. The basis of our strategy was the find, organize, clarify, understand, select-plan, do, check, and act (FOCUS-PDCA) model, developed by engaging key stakeholders, including patients. More than 300 healthcare professionals and patients from around the Riyadh region assessed the current system with three solutions and roadmap workshops. Subsequently, the national clinical advisory group (CAG) for trauma was formed to develop the Saudi Arabian trauma system, and CAG members analyzed and collated internationally recognized trauma systems and guidelines. The guidelines’ applicability in the kingdom was discussed and reviewed, and an interactive document was developed to support socialization and implementation. The CAG team members agreed on the guiding principles for the trauma pathway, identified the challenges, and finalized the new system design. They also developed a trauma care standard document to support and guide the rollout of new trauma networks across the kingdom. The CAG members and other stakeholders are at the forefront of implementing the trauma system across the Riyadh region. Recent trauma system development in Saudi Arabia is the first step in improving national trauma care and may guide development in other locations, regionally and internationally, to improve outcomes.

## Introduction

Worldwide, approximately five million people lose their lives every year due to physical injuries. It constitutes about 9% of the world’s total death ratio and represents 1.7 times the number of mortalities resulting from HIV/AIDS, tuberculosis, and malaria combined. The leading causes are violence (homicide and suicide), motor vehicle crashes, falls, burns, drowning, and poisoning. By 2030, motor vehicle crashes alone are predicted to become the 7^th^ leading cause of death worldwide [1]. Effective trauma care can be explained as an incorporated, protocol-driven system of care that addresses the complete trauma care pathway, including prehospital emergency medical care, in-hospital management, rehabilitation, and trauma prevention. The World Health Organization sponsors various activities to reduce injury-related morbidity and mortality [2], including surveillance and basic research through prevention programs and effective strategies for trauma management. Much importance has been placed on preventive measures, such as seatbelts, traffic rules, speed limits, and enforcing the prohibition of driving under the influence (e.g., alcohol breath-testing). There are also significant gains to be made by improving the management of trauma patients, especially in the prehospital arena and initial management upon arrival at the hospital [3, 4].

The trauma care system can be inclusive or exclusive. In the inclusive approach, injured patients are managed across the network by organizing the healthcare facilities within the region, and in an exclusive system, patients are transferred directly and managed in a tertiary hospital that is well equipped for trauma management [5]. The implementation of a trauma system can reduce mortality by up to 15% [6–10]. In the Kingdom of Saudi Arabia (KSA), the major reason for death is trauma in the first four decades of life, and it is also the leading cause of disability among young and productive members of Saudi society. The incidence of significant trauma and associated fatalities in Saudi Arabia has increased in recent years with population growth, increased vehicle ownership, and the rapid development of highway infrastructure [11, 12]. Motor vehicle crashes (MVCs) are the second-highest cause of mortality for men and children, with incidents rising by 8.5% from 2005 to 2016 [13, 14]. Between 2001 and 2010, the most common type of injury was due to MVCs (52.0%), followed by falls (23.4%), with MVCs resulting in 7,661 fatalities in 2013 (88% M, 12% F) [15, 16]. In the Riyadh region in 2017, there were over 27,000 MVC-related emergency admissions and over 120,000 non-MVC-related trauma admissions, emphasizing the scale of the problem [16].

The post-crash care of trauma patients poses a significant challenge. Prompt delivery of severely injured patients to a hospital that can provide the most appropriate care in the least time improves chances of survival [13, 17–19]. The civilian population of the KSA currently lacks access to an organized trauma system that recognizes the complexity, range, and time-critical nature of injuries requiring immediate, integrated care by dedicated trained personnel at specialized trauma centers. Specific challenges, such as vast distances, harsh climate, and limited communication infrastructure, contributed to complications in this regard. This article aims to describe the design, development, and initial adoption of the Saudi Arabian trauma system, in line with Vision 2030.

## Material and Methods

**Figure 1.**
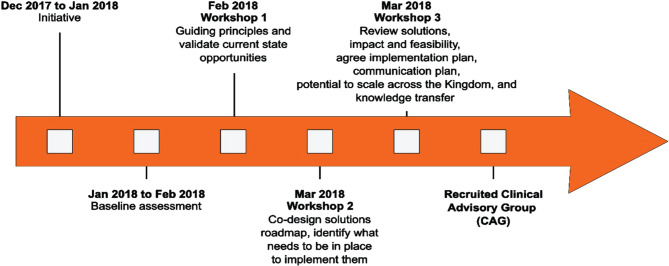
Design phase overview.

The base of our plan was developed by engaging key stakeholders, including patients. More than 300 healthcare professionals and patients from the Riyadh region assessed the current system in three solutions and roadmap workshops using the find, organize, clarify, understand, select-plan, do, check, and act (FOCUS-PDCA) methodology [20]. Subsequently, a national clinical advisory group (CAG) for trauma, under the supervision of the Vision Realization Office (VRO) of the Ministry of Health (MOH), was formed to develop the Saudi Arabian trauma system (Figure 1).

The CAG for trauma was a team of 11 members consisting of experts from various fields, including emergency medical services (EMS), emergency and disaster management, emergency department, trauma surgery, nursing, pediatric emergency, trauma research, and rehabilitation (Table 1). The CAG members set up the national standard for trauma care.

**Table 1.** CAG members.

**Member**	**Position and affiliation**
**Dr. Thamer Nouh**	CAG chairman, associate professor of surgery, College of Medicine, King Saud University, Riyadh
**Dr. Sharfuddin Chowdhury**	Trauma surgeon, director of trauma center, King Saud Medical City, Riyadh
**Dr. Ahmed Alburakan**	Assistant professor of surgery, College of Medicine, King Saud University, Riyadh
**Dr. Talal Altahan**	Head of the Department of Surgery, Prince Mohammed bin Abdulaziz Hospital, Riyadh
**Dr. Ibrahim Albabtain**	Director of Trauma Research Program, King Abdulaziz Medical City, Riyadh
**Dr. Jalal Alowais**	Supervisor general of the General Directorate of Emergency, Disaster and Ambulance Services, MOH, Riyadh
**Ms. Jehad Hassan Alalshikh**	Emergency nurse, King Abdulaziz Medical City, Riyadh
**Mr. Abdulaziz Alotaibi**	Chief of Rehabilitation Services, King Abdulaziz Medical City, Riyadh
**Mr. Nasser Almadhi**	Emergency health services specialist, emergency services Project Manager, MOH, Riyadh
**Dr. Mohammed Azzam**	Emergency medicine consultant, Dr. Soliman Fakeeh Hospital, Jeddah
**Dr. Ahmed Muneer Althekair**	Pediatric emergency medicine consultant, Prince Sultan Military Medical City, Riyadh

The approach adopted was to analyze and collate internationally recognized trauma systems and guidelines, and a thorough review was undertaken by the CAG members. Further analysis workshops and discussions on those guidelines and their applicability in the kingdom were held. These were shared with cluster clinical colleagues for review, and an interactive document to support socialization and implementation was developed. The guiding principles for pathway development are described in Table 2. These concepts helped guide the transformation trajectory.

**Table 2.** The guiding principles.

**Value-based healthcare**	Investments in healthcare solutions should be made to maximize patient outcomes and financial sustainability in the short, medium, and long term.
**Equity of access**	All patients should be treated equally and receive timely, effective care, regardless of status, location, or wealth.
**Innovative thinking**	The pathway should be created using innovative ideas from colleagues and patients.
**Networked staff/institutions**	Collaboration between ministries, providers, and organizations is essential for the pathway’s success.
**Patient first**	Patient safety, outcomes, and needs should be considered at the heart of every decision.
**Best clinical practices**	The care provided should be standardized and based on international evidence. Data should be collected to allow for continuous improvement, knowledge sharing, auditing, and transparency across the system to ensure best practices are being applied.

## Results

The CAG team members agreed on the trauma pathway’s fundamental guiding principles, identified the challenges, and finalized the new trauma care system’s design. They also developed a trauma care standard document to support and guide the rollout of new trauma networks across the KSA. The CAG members and other stakeholders are also at the forefront of implementing the trauma system across the Riyadh cluster. The trauma pathway development, trauma care standard documents, and adoption of regional trauma systems are described below.

### A. Trauma pathway development

During the workshop, the trauma system’s four pillars-prevention, prehospital, in-hospital, and rehabilitation – were discussed thoroughly to identify the gaps and areas. Six areas of challenges were identified: prehospital, resource control center, hospitals, rehabilitation and community discharge, rural trauma care, and pediatric trauma care. The deficits in these areas are shown in Table 3.

**Table 3.** Six areas of challenges and gaps.

**Areas of Challenge**	**Gaps**
**Prehospital (hotline + transport)**	Transport resources and capabilities
**Resource Control Center**	No collaborative networks
**Hospitals**	Lack of transportation
**Rehab and community discharge**	Number of staff and public awareness
**Rural trauma care**	Geographic networks
**Pediatric trauma care**	Hospitals

Proposed solutions to current challenges were 1) appropriate allocation of resources; 2) development of standards and guidelines; 3) establishment of clinical advisory group; 4) development of efficient prehospital transportation; 5) development of rehabilitation and early treatment of disease; 6) development of telemedicine and technology; 7) standardization of training and care across the system, and 8) development of a peer-supported clinical network.

**Figure 2.**
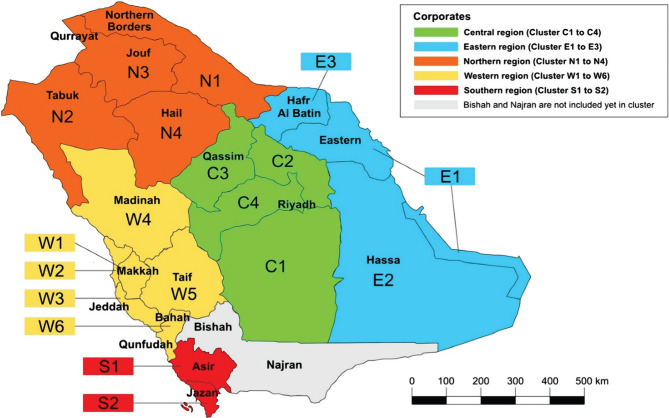
Geographical region and cluster map for health care services in the KSA.

Subsequently, the CAG for trauma began their work in the Riyadh region. The KSA is divided geographically into 19 clusters, not including Bishah and Najran in the south (Figure 2).

**Figure 3.**
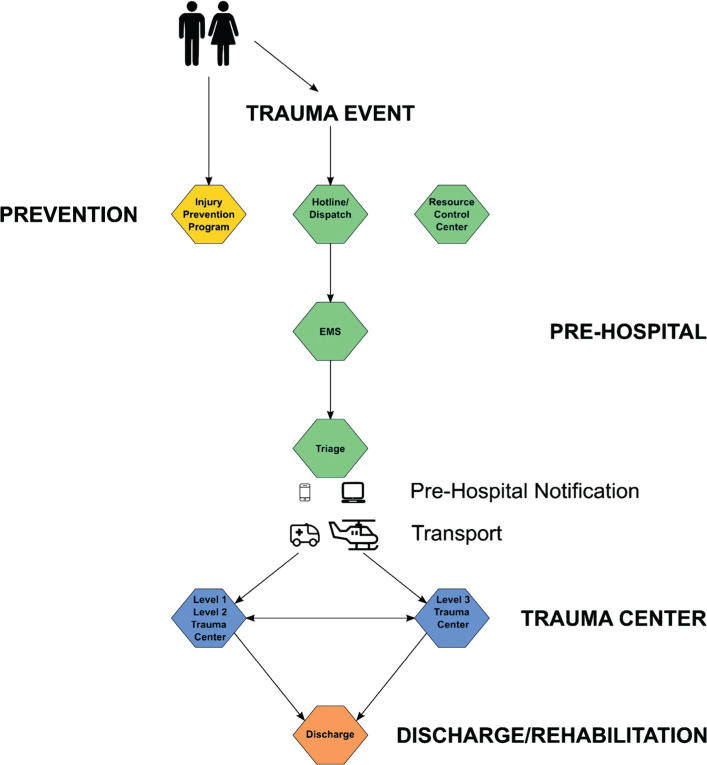
Trauma pathway.

The Riyadh region is divided into four clusters (C1–C4). Each cluster will serve as a complete unit for trauma care in Riyadh’s regional trauma network and will have a level 1 trauma center, at least two level 2 trauma centers, and multiple level 3 trauma centers, as per CAG standards. After a trauma event, EMS will be dispatched to the scene through a hotline (997). After triaging, EMS will send a prehospital notification to the appropriate level of trauma center regarding patient arrival. Prehospital activity, as well as interhospital transfer, will be coordinated, regulated, and monitored by the central resource control center. After managing patients in the hospitals, they will either be discharged or sent to home care facilities or short- or long-term rehabilitation facilities. The trauma pathway for each cluster is shown in Figure 3.

### B. Development of trauma care standard document

The CAG formulated the trauma care standards document to support and guide the rollout of new trauma networks across the kingdom. It will provide a framework that will support the development and implementation of dedicated trauma services using fit-for-locality networked provider models across all regions of the KSA. These trauma service standards will help health system administrators, trauma networks, and service providers judge their local services’ quality and plan for improvements as needed. The document is designed for all health care professionals, including administrators.

A range of national and international guidelines, standards, and evidence was reviewed, and expert opinions from CAG members were used to develop these standards for the KSA [2, 21, 22]. While the document sets an optimal endpoint, there are still areas where further recommendations, including clinical care standards for the management of specific traumatic injuries, are required. The standards are geared toward ensuring optimal care for injured patients within acute care facilities specializing in trauma care.

According to these standards, Saudi trauma centers will be at three levels. A level 1 trauma center should be capable of emergency and definitive complex trauma management in the presence of all subspecialty facilities. It will also provide teaching, research, leadership, and outreach programs. A level 2 trauma center will involve emergency trauma reception, resuscitation, and emergency surgical management to save life or limbs. Surgical specialties such as general surgery, orthopedic surgery, neurosurgery, and critical care services are mandatory, and other specialized care is desirable at this trauma center level. A level 3 trauma center will evaluate, assess, and stabilize injured patients before onward transfer to higher-level centers, based on agreed transfer thresholds and protocols.

The document describes the minimum criteria for designation and approval for each level of a trauma center in detail, as well as administrative roles and the formation of trauma committees at different levels, such as regional, network, and cluster levels. The detailed service standards for different specialties involved in trauma management, policy protocol, clinical pathway guidelines, e.g., long-term care and transfer, and performance improvement indicators are also delineated in the document. The VRO at MOH transferred responsibility for this standard document to the Saudi Central Board for Accreditation of Healthcare Institutions (CBAHI) for the designation and accreditation of a trauma center. The CBAHI trauma center certification requirements are described in Table 4.

**Table 4.** CBAHI trauma center certification requirements.

Program participation requirements
• The original healthcare setting is accredited by CBAHI. • The healthcare organization’s infrastructure/official agreements enable the surgical, medical, and rehabilitative management of trauma victims. • The program is active for at least six months before the certification accreditation visit and has managed at least (number to be determined) cases of trauma (types and numbers according to levels).
Program design and management
• Program leaders ○ Appropriate certification, training, and experience ○ Roles and responsibilities • Mission and vision ○ Scope of services ○ Strategic planning ○ Other service participation ○ Admission and discharge criteria (will determine the center level)
Provision of care
• Access to care • Essential requirements for services ○ Staffing levels ○ Staff training and competency requirements ○ Structural requirements of the hospital ○ Essential equipment ○ Assessment/reassessment ○ Essential investigations ○ Turnaround times for investigations ○ Turnaround times for consultations ○ Care planning and consultations ○ Practice guidelines ○ Essential support services ○ Outcome measures
Quality improvement, risk management, and patient safety program
• Program design • Program management and staffing • Performance measurement • Reporting of events and near misses • Resource utilization • Information management

### C. Adoption of regional trauma system

**Figure 4.**
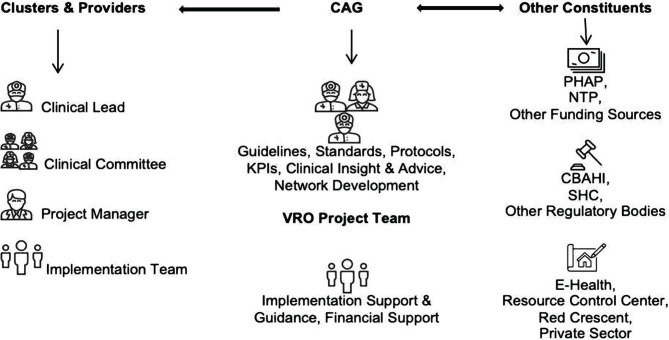
Approach for collaboration and team for implementation. CAG – Clinical Advisory Group; VRO – Vision Realization Office; PHAP – Program of Health Assurance and Purchase of Health Services; NTP – National Transformation Program; CBAHI – Central Board for Accreditation of Healthcare Institutions; SHC – Saudi Health Council.

After setting up the trauma care pathway, it was a great challenge to implement it cluster-wise and regionally. Therefore, we followed an inclusive, collaborative approach and involved all stakeholders. The strategies for collaboration and teams for implementation are shown in Figure 4. The progress of implementing the trauma system in the Riyadh region until December 2019 is shown in Table 5.

**Table 5.** Trauma system implementation progress (until December 2019).

**Key Planned Milestones**	Status
**Readiness assessment, gap analysis, and hospital classification**	Completed for C1 and C2. Not started in N3.
**Implementation plan**	Completed for C1 only. Not started in C2 and N3.
**Network clinical committee**	Established for C1 only. C2 and N3 are in progress.
**Adequate cluster PMO support**	Yes, for C1 and N3. C2 is in progress.
**Socialization of standards/guidelines**	C1 and N3 are in progress. Not started in C2.
**Clinical registry use**	C1 is in progress. Not started in C2 and N3.
**The first patient on the pathway**	Completed in C1. Not started in C2 and N3.
**Network-based working**	C1 is in progress. Not operational in C2 and N3.

C1 – Cluster 1; C2 – Cluster 2. We have used N3 for King Khaled University Hospital, as it is not in a cluster, but aims to develop a clinical network C3. PMO – Project Management Office.

## Discussion

This report details the significant advances in realizing a trauma system in Saudi Arabia, providing a firm basis for further developing an integrated trauma system. The design phase helped understand the key current state issues, guiding principles for future state design, priorities for implementation, and a draft pathway for trauma. The magnitude of trauma in Saudi Arabia has recently been reduced by primary prevention strategies that attempt to modify the behavioral and environmental factors that lead to injury. The government has implemented prevention programs, such as increased seat belt usage, speed limit enforcement, speed camera surveillance, and workplace safety reforms, which have resulted in a decline in trauma death rates in some cities in Saudi Arabia [23]. Current evidence suggests that a network-based approach providing an effective nationwide trauma care coordination system is the best approach for managing trauma emergencies and improving outcomes. In response to the burden of disease and to fulfill the goals of Vision 2030, under the national transformation program, the VRO of the MOH created the CAG to establish and improve urgent care pathways in the kingdom, including trauma, stroke, and acute coronary syndrome.

In the West, trauma care is better established. The differences between trauma care systems in developing and developed countries are vast. Even in countries with optimum trauma care, there are variations in regional-level organization. An excellent example of such variations can be identified in European Union (EU) states, where there are no current unified standards of trauma care. Each country has its own method for organizing prehospital care, trauma teams, and rehabilitation, and trauma surgeon training standards are also different. In Austria, there is an isolated department for trauma surgery, while trauma surgery is integrated with other specialties in other EU countries, such as orthopedic surgery in Switzerland and Belgium and general surgery in the Netherlands and Italy. Another variation is found in Britain, France, Germany, Portugal, and Scandinavian countries, where trauma care is covered by different specialties, each with its own area of responsibility [24, 25]. Overall, trauma care seems more established in England and Central European countries than in Wales, Scotland, Northern Ireland, Scandinavia, and Mediterranean countries [25].

The efficiency of trauma systems in reducing death from injury was most prominently documented in the United States (US). Its effectiveness is such that in 1990, the US Congress approved the Trauma Care Systems Planning and Development Act, requiring states to develop trauma care systems. One study found that, on average, mortality due to traffic accidents was reduced by 8% (95%, CI 3%–12%) 15 years after implementation [26]. International evidence shows that the odds of a significant trauma patient’s survival can be improved by 63% through the strategic provision of major trauma centers in or close to major population centers. In the US, a study of the 25 largest metropolitan areas in 19 states found that regionalization of care to specialist trauma units decreases mortality by around 25% and stay length to four days [27]. Another US study also found that trauma treatment at a trauma center versus a non-trauma center protected over 3.4 lives per 100 patients treated at a fiscally convenient ratio of $36,000 per quality-adjusted life-year gained [28].

The American College of Surgeons Committee on Trauma (ACS-COT) classifies, designates, and accredits trauma care amenities within a trauma system into five levels (I–V) in the US [22]. In a few states, the state government runs the evaluation process, and some states classify every acute hospital according to the level of care it can deliver. Other states provide trauma care through a limited number of designated level 1 and level 2 trauma centers [29].

Mainland Australia has national and statewide trauma care systems, with designated trauma centers in New South Wales that started in the early 1990s and are still evolving. The trauma network in Australia is divided into metropolitan and rural areas, with three levels in each network. Level 1 (major trauma service) offers a complete range of trauma care in the metro trauma network and acts as a tertiary referral center. It is also a center for trauma research and education and plays a leading role in providing state-of-the-art trauma care in the region. A level 2 urban trauma service delivers preliminary evaluation and stabilization and, when justified, initiates transfer to a level 1 trauma center. The level 3 metropolitan trauma service provides special attention for patients with even minor injuries in the local communities in urban areas.

In the rural trauma network, first-level (regional trauma services) offer definitive care to nonmajor trauma patients according to the hospital’s availability of expertise. At the second level, rural trauma services have 24-hour on-duty medical practitioners to deliver trauma care. Remote rural trauma services are at the third level, providing trauma care in remote area hospitals with no immediately available general practitioners [30].

## Conclusions

Trauma care systems are a structured, multidisciplinary response to injuries and their prevention through a continuum of care that aims to return those injured to their pre-injury state. They are a network of organizations working together in a geographic area to plan, provide, and manage injuries in all aspects of trauma care, from injury prevention to rehabilitation. A uniform network-based method for all acute care facilities to deliver reliable, high-quality care to the injured is essential in any health system. Recent trauma system development in Saudi Arabia is the first step in improving national trauma care. It may guide development in other locations, regionally and internationally, to improve outcomes.

## Acknowledgments

### Conflict of interests

The authors declare that there is no conflict of interest.

### Authorship

The study was conceptualized and designed by SC and LL. SC and DM collected and analyzed pertinent data. SC wrote the manuscript. SC and DM created images. The paper was subjected to a critical evaluation and appropriate modification by LL. The final manuscript was read and approved by all authors.
